# Physical Properties of Starches Modified by Phosphorylation and High-Voltage Electrical Discharge (HVED)

**DOI:** 10.3390/polym14163359

**Published:** 2022-08-17

**Authors:** Đurđica Ačkar, Marijana Grec, Ivanka Grgić, Artur Gryszkin, Marzena Styczyńska, Antun Jozinović, Borislav Miličević, Drago Šubarić, Jurislav Babić

**Affiliations:** 1 Faculty of Food Technology Osijek, Josip Juraj Strossmayer University of Osijek, F. Kuhača 18, 31000 Osijek, Croatia; 2 Institute of Public Health Brod-Posavina County, V. Nazora 2A, 35000 Slavonski Brod, Croatia; 3 Department of Food Storage and Technology, Faculty of Biotechnology and Food Science, Wrocław University of Environmental and Life Sciences, Chełmońskiego 37, 51-630 Wroclaw, Poland; 4 Department of Human Nutrition, Faculty of Biotechnology and Food Science, Wrocław University of Environmental and Life Sciences, Chełmońskiego 37, 51-630 Wroclaw, Poland; 5 Polytechnic in Požega, Vukovarska ulica 17, 34000 Požega, Croatia

**Keywords:** starch, HVED, phosphorylation, pasting properties, swelling power, gel texture

## Abstract

High-voltage electrical discharge (HVED) is considered as a novel, non-thermal process and is currently being researched regarding its effect on microorganisms (decontamination of food), waste water treatment, and modification of different compounds and food components. In this paper, four native starches (maize, wheat, potato, and tapioca) were treated with HVED, phosphorylated with Na_2_HPO_4_ and Na_5_P_3_O_10_, and modified by a combination of HVED with each phosphorylation reaction both prior and after chemical modification. Pasting properties, swelling power, solubility, gel texture, and particle size were analyzed. Although HVED induced lower contents of P in modified starches, it had an effect on analyzed properties. The results revealed that HVED treatment alone had a limited effect on pasting properties of starches, but it had an effect on properties of phosphorylated starches, both when it was conducted prior and after the chemical modification, reducing the influence of Na_5_P_3_O_10_ and Na_2_HPO_4_ on the decrease of pasting temperature. With minor exceptions, the gel strength of starches increased, and the rupture strength decreased by all modifications. HVED treatment resulted in a decrease of the particle size after the modification of maize and wheat starches, while potato and tapioca starches were not significantly influenced by the treatment.

## 1. Introduction

Starch is an abundant raw material extensively used in the food industry, pharmacy, paper industry, etc. It has gelling, thickening, and binding properties, and is cost-effective, and these are the major reasons for its broad use. However, a native starch, regardless of the origin, does not have ideal properties desired for specific uses. Therefore, it is often modified by chemical, enzyme, and/or physical procedures. The most commonly used techniques are esterification (such as acetylation) and cross-linking (with epichlorohydrin, adipate or phosphate). It is well established that esterification of starch results in decreased gelatinization temperatures, higher paste clarity, starches are less prone to retrogradation. Cross-linking will bring about thicker pastes that are more resistant to shearing at high temperatures. Although starches with properties suitable for food- or pharmaceutical industry are obtained, these processes are time-consuming, require special attention due to chemical disposal, and often require heating. Therefore, nowadays, more environmentally friendly and energy-efficient methods are being explored. Fan and Picchioni [1] gave the review of more recent methods used in modification of starch, such as use of green solvents, regioselective derivatization, and transfer radical polymerization. Although the number of research on these subjects is limited, they are promising, with respect to lower environmental impact and the potential to produce tailor-made starches for specific application. However, the authors of the review conclude that there is still a lot to be done in these fields of research. Along with methods listed by Fan and Picchioni [1], high-voltage electrical discharge could be a promising technique.

High-voltage electrical discharge (HVED) is an emerging technology in food processing. It is already well explored for an application in extraction processes and decontamination of food [2,3,4], but it could have a potential in the starch modification as well [5].

Namely, a high-voltage discharge between two electrodes submerged in the water causes a high-energy release, an ionization of water molecules, a formation of free radicals and bubbles. It affects compounds by both the physical damaging and chemically induced changes. This makes it applicable in the starch modification.

Phosphorylation is the only naturally occurring covalent modification of starch [6]. In cereal starch, starch-bound phosphate is usually present in traces, and amounts of 0.2–4.4% *w*/*w* in potato starch have been reported. Phosphate groups in native starch are bound to C-6 and C-3 positions of glucose units of amylopectin in the form of monoester [6,7]. Hydration capacity after gelatinization is increased by the presence of phosphate groups [6], they enable ion-exchange, gel forming and complexing actions [7], and influence digestibility of starch [6,7], which is a ground for chemical modification of starch by phosphorylation for industrial purposes [6].

Phosphorylated starches may be mono- or distarch phosphates. Monoesters are usually prepared using sodium tripolyphophate or mixtures of sodium dihydrogen phosphate dihydrate and disodium hydrogen phosphate dehydrate, whereas cross-linked starches (distarch phophates) are commonly prepared using phosphorous oxychloride or sodium trimetaphosphate. Cross-linked starches contain lower contents of P in relation to esterified starches (0.04% in relation to 0.1–0.4%) [7]. Mechanisms of starch phosphorylation by different chemical reagents are given in the review by Ramadan and Sitohy [7]. In addition, reaction mechanism of phosphorylation by Na_5_P_3_O_10_ on C-6 is proposed by Li et al. [8] and by Na_2_HPO_4_ is proposed by Ramdan and Sitohy [8], whereas molecular models of phosphorylated starch at C-3 and C-6 are proposed by Blennow et al. [6].

In our previous paper [9], we explored the influence of HVED and its combination with phosphorylation on gelatinization properties, the starch damage, and the content of resistant starch. This paper focuses on pasting properties, swelling power and solubility, gel texture and particle size, which are important properties for the starch application in the food production.

## 2. Materials and Methods

Maize, potato, and tapioca native starches were supplied by Cargill, USA, and the wheat starch was isolated at the Faculty of Food Technology Osijek, as previously described [10]. All starches are food-grade. Na_2_HPO_4_ (p.a.) and Na_5_P_3_O_10_ (p.a.) were products of Acros Organics (Geel, Belgium).

### 2.1. Modification of Starch

Starches were modified as described in the previous paper [9]. Briefly, for the high-voltage electrical treatment (HVED), 200 mL of 1 g/mL (d. m. b.) of a starch suspension was prepared and treated with the custom-made HVED device (30 kV, 70 Hz, 30 min), with stirring at magnetic stirrer. The HVED device is described in more detail in Barišić et al. [11]. The modification of starches with Na_5_P_3_O_10_ was done according to the procedure described by Lim and Seib [12], and with Na_2_HPO_4_ according to Choi et al. [13] and Prasanthi and Rama Rao [14] with slight modifications. Briefly, starch (100 g of d.m.) and Na_2_HPO_4_ were suspended in 200 mL of demineralized water with stirring at magnetic stirrer for 30 min. The suspension was centrifuged and starch was air-dried overnight, and then treated in the oven at 130 °C, washed three times with water, and dried until moisture <85% was achieved. Combined treatments were done both by first treatment with HVED and subsequently with one of the chemical modifications and vice versa [9].

### 2.2. Phosphorus Content

The sample was prepared as described in PN-EN ISO 3946: 2000 [15], and phosphorus was determined by inductively coupled plasma–optical emission spectrometry (ICP–OES).

In brief, the samples were digested “wet” in a closed microwave system with 5 cm^3^ of concentrated nitric acid (V) p.a. and 1 cm^3^ of concentrated hydrogen peroxide p.a., then the samples were mineralized in the microwave MARS 5 (CEM, Matthews NC, USA) sample preparation system. The minerals were quantitatively transferred to 10 cm^3^ measuring vessels with re-distilled water. An appropriate amount was taken for the determination of phosphorus.

Phosphorus was determined by induced plasma atomic emission spectrometry-ICP-OES using the ICP-AES iCAP 7400 atomic emission spectrometer (Thermo Scientific, Waltham, MA, USA). The results were confirmed using the certified reference material NCS ZC 73012-Cabbage, and the measurement uncertainty was estimated at 5%.

### 2.3. Pasting Properties

The pasting properties of starches were determined by the Brabender micro visco-amylograph (Brabender GmbH., Duisburg, Germany). A starch was suspended in distilled water (7% d.w.) and subjected to the following temperature program: heating to 92 °C (7.5 °C/min), holding at 92 °C for 15 min, cooling to 50 °C (7.5 °C/min), and holding at 50 °C for 15 min. The measuring was done at 250 rpm.

### 2.4. Swelling Power and Solubility

The swelling power (SP) and the solubility (SOL) were determined by heating 1% (d. m.) starch suspension at a pre-set temperature (65, 75, 85, or 95 °C) for 15 min in the shaking water bath (Julabo SW22, Julabo GmbH, Seelbach, Germany) with subsequent cooling and centrifuging (IEC Centra MP**4R**; 3000 rpm, 10 min). The supernatant was decanted and used to determine the dry matter content (105 °C until reaching constant mass) and the gel was weighed.

The swelling power (SP) was calculated according to Equation (1), and the solubility (SOL) according to Equation (2):

SP = WG/Wd.m.G(1)

where: SP (g/g) is the swelling power; WG (g) is the mass of gel; Wd.m.G (g) is the mass of the dry matter in gel.

SOL = (Ws/W0) × 100(2)

where: SOL (%) is solubility; Ws is the dry matter content in the supernatant; W0 is the dry matter content in the suspension (1%).

### 2.5. Texture Properties

Starch suspensions (11% d.w.) were gelatinized in the shaking water bath (Julabo SW22) at 95 °C for 30 min and allowed to gel. The gel formed in cups (35 g of suspension poured in cups with 35 mm diameter, 50 mm height) and was compressed with the flat cylindrical probe (20 mm diameter) in the TA.XT Plus (Stable Mycrosystems, Surrey, UK) at the speed of 2.0 mm/s to the distance of 20 mm. The peak height at 20 mm compression was termed hardness, and the negative area of the curve during retraction of the probe was termed adhesiveness. The distance at break was termed brittleness and the force, expressed in grams, was necessary to depress the surface gel strength by 4mm.

### 2.6. Particle Size

The particle size was determined by the Masterizer 2000 (Malvern Instruments LTD, Malvern, UK) with Hydro 2000 MU adapter at 20 °C, and the obscuration between 15 and 20%. Before each measurement, starch agglomerates were disintegrated by the ultrasound with a frequency of 10 Hz for a period of 10 s.

### 2.7. Statistical Analysis

All analyses were done in triplicates and the obtained results were statistically analyzed by analysis of variance and Fischer LSD test in Statistica^®^ 13 (*p* < 0.05). Results are expressed as a mean value ± standard deviation.

## 3. Results and Discussion

In order to explore applicability of the high-voltage electrical treatment (HVED) in the starch phosphorylation, four starches—maize, wheat, potato, and tapioca—were treated with the HVED alone, phosphorylated alone with Na_5_P_3_O_10_ or Na_2_HPO_4_, and modified by the combination of HVED with phosphorylation. Phosphorus content, pasting properties, swelling power, solubility, gel texture, and particle size were determined in order to evaluate the influence of the afore mentioned modifications on the starch properties.

**Figure 1:**
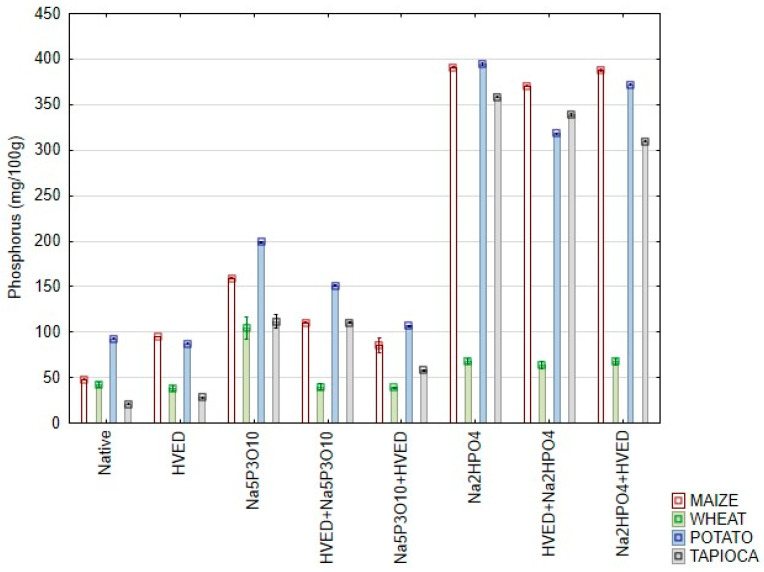
Phosphorus contents (mean ± 2SD) in starches modified with high-voltage electrical discharge (HVED) and phosphorylation.

The contents of phosphorus in analyzed samples are shown in Figure 1. It is well established that starch naturally contains low content of phosphorus, linked to C-2 and C-3, and in form of phospholipids [16]. In the present research, contents of 20.82–92.86 mg P/100 g starch was determined, depending on type of starch. HVED treatment resulted in elevated values of P contents in maize and tapioca starch, and lower values were determined in wheat and potato starch. Due to the complexity of a HVED process, different reactions may have occurred in investigated samples: extraction in the water, formation of complexes in which P could be “masked” and available during determination. Namely, Du et al. [17] reported that OH and O_2_ formed by HVED reacted with aromatic ring, resulting in ring-cleavage products, and Grinevich et al. [18] reported a decrease of Pb, Cd, and Mn in wastewater after HVED treatment, showing the influence of the treatment both on organic and inorganic compounds and different mechanisms of reactions.

Both phosphorylation agents increased contents of P in starches, an indication that the reactions of phosphorylation were successful. In all cases, except the wheat starch, higher contents of P were determined in starches modified with Na_2_HPO_4_. Lower contents of P in starches modified by combination of HVED with phosphorylating agents showed that HVED treatment slightly reduced the efficiency of reaction, regardless the agent used. However, HVED induced changes of starch properties when combined with chemical modification, as shown both in our previous [9] and present research.

Pasting properties of native and modified starches are shown in Table 1. While the HVED treatment did not significantly influence the pasting temperature of wheat and tapioca starches, it resulted in the decrease of the pasting temperature of maize starch and its increase for potato starch. Starches with higher crystalline order paste at higher temperatures [19,20] and this shows that the HVED treatment disrupted the crystalline order of the maize starch, while in the potato starch it caused the opposite effect. Potato starch granules are very large and characterized by a B-type of granules, which contain much more water than an A-type granules typical for maize starch [21]. Water molecules inside granules could have also been excited by the HVED and could have initiated reactions of cross-linking in the potato starch [22,23]. On the other hand, HVED has been shown to induce the formation of fissures and cavities [24], the enlargement of channels, and the partial fracture of granules [22]. This may have caused disordering of the high crystalline order of the maize starch, which is supported by the increase of its maximum viscosity and viscosities at 92 and 50 °C (Table 1).

Wheat starch has both A- and B-type granules and opposite effects may have nullified each other, whereas C-type granules of tapioca starch, although not well studied yet, probably have amorphous region in the inner part of granule [25], which may be the reason for its resistance to HVED. Interestingly, the breakdown value was significantly influenced by the HVED only in the maize starch, showing that it became more susceptible to the shearing at high temperatures. This is consistent with the rise of peak viscosity, which is often followed by a rapid loss of viscosity [26]. Increase of viscosity after treatment with a low pressure radio frequency plasma was observed by Banura et al. [27] for corn and tapioca starches, while Wu et al. [28] reported a decrease of viscosities of banana starch after the treatment with corona electrical discharge.

**Figure 2:**
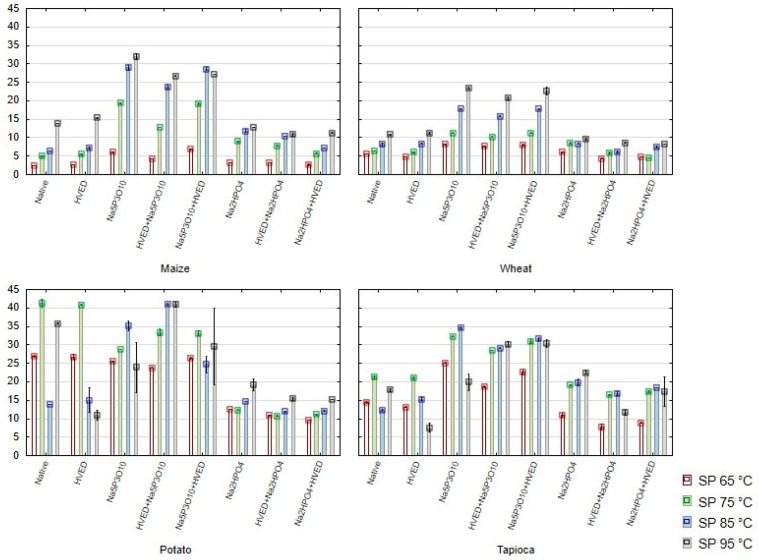
Swelling power (SP) (mean ± 2SD) of starches modified with high-voltage electrical discharge (HVED) and phosphorylation.

The phosphorylation with Na_5_P_3_O_10_ reduced pasting temperatures of all investigated starches, whereas Na_2_HPO_4_ induced the increase of the pasting temperature of the tapioca starch. Na_5_P_3_O_10_ resulted in the significant increase of peak and viscosities at 92 °C and 50 °C. All these indicate a larger absorption of water and swelling of granules, supported by the results for swelling power shown in Figure 2. Introduction of large substituents causes spacing between starch chains and eases a penetration of water between them. The same effect was reported by Ascheri et al. [29] for phosphorylated wolf’s fruit starch and by Nathania et al. [30] for phosphorylated mung bean starch.

The HVED treatment reduced an effect of Na_5_P_3_O_10_ and Na_2_HPO_4_ on the decrease of pasting temperature in all samples, except for the wheat starch in combination with Na_5_P_3_O_10_, where it additionally reduced pasting temperature. Generally, it may be observed that the HVED treatment combined with Na_5_P_3_O_10_ increased the viscosity compared to native starches, while in the combination with Na_2_HPO_4_ it caused the increase of hot and cold viscosities for maize and the decrease of viscosities for potato, tapioca, and wheat starches. With the exception of the maize starch, a marked effect on the breakdown and setback values was observed for starches treated with the combination of HVED and Na_2_HPO_4_, showing that viscosities of these samples are more stable, during both shearing at high temperatures and cooling.

A marked influence on the swelling power (SP) of starches is observed only at higher temperatures (85 and 95 °C) for phosphorylated starches, both with and without combination with the HVED (Figure 2). While Na_5_P_3_O_10_ increased the SP, Na_2_HPO_4_ generally decreased it. Some deviations were observed for potato and tapioca starches, but this was probably caused primarily due to their high solubility and a difficult separation of the gel from the supernatant during the analysis. Nathania et al. [30] also reported the increase of swelling power and solubility after treatment of mung bean starch with Na_5_P_3_O_10_.

**Figure 3:**
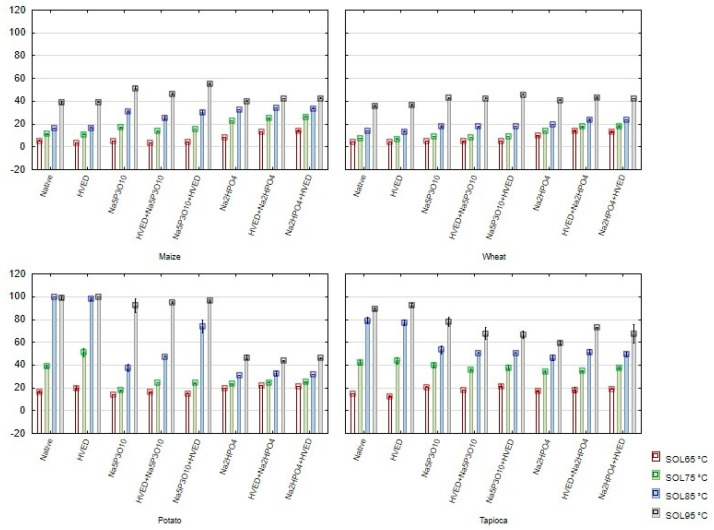
Solubility (SOL) (mean ± 2SD) of starches modified with high-voltage electrical discharge and phosphorylation.

The solubility (SOL) of maize and wheat starches was not affected significantly by any modification, while at higher temperatures, solubility of the potato starch modified with Na_2_HPO_4_ both with and without combination with the HVED markedly decreased (Figure 3). A decreased solubility of phosphorylated tapioca starches, both with and without combination with HVED, was observed at 95 °C.

Wongsagonsup et al. [31] stated that swelling power and solubility depend on the extent of cross-linking induced by phosphorylation—at a lower level of cross-linking water penetration is easier and more starch granules leach into the solution, while at higher levels of cross-linking water penetration is hindered and leaching of polymers is reduced.

**Figure 4:**
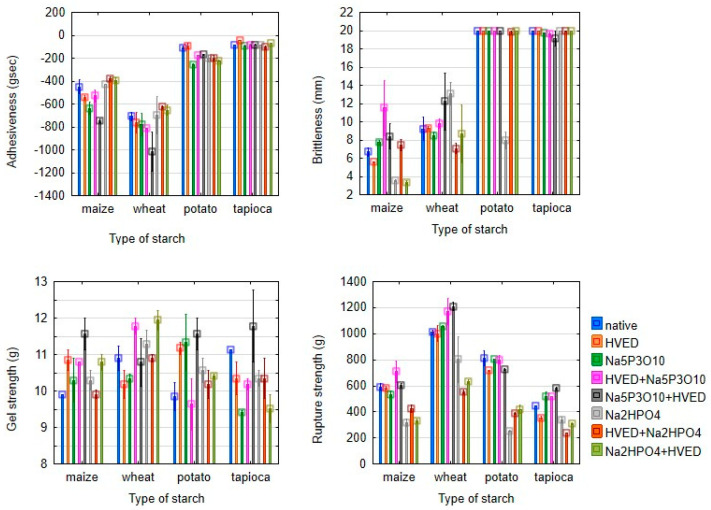
Gel texture properties (mean ± 2SD) of starches modified with high-voltage electrical discharge and phosphorylation.

Gel texture is another property of starch relevant for its practical use. Adhesiveness and brittleness of potato and tapioca starches were not significantly influenced by modifications (Figure 4). The HVED treatment and the modification with Na_5_P_3_O_10_ both with and without combination with HVED reduced the adhesiveness of maize and wheat starches. With minor exceptions, the gel strength of maize, wheat, and tapioca starches increased, and the rupture strength decreased by all modifications. The increase of gel strength was observed for the wheat starch modified with succinic acid/acetanhydride mixture, and the decrease of adhesiveness was observed for the acetylated tapioca starch in our previous research [32,33].

**Figure 5:**
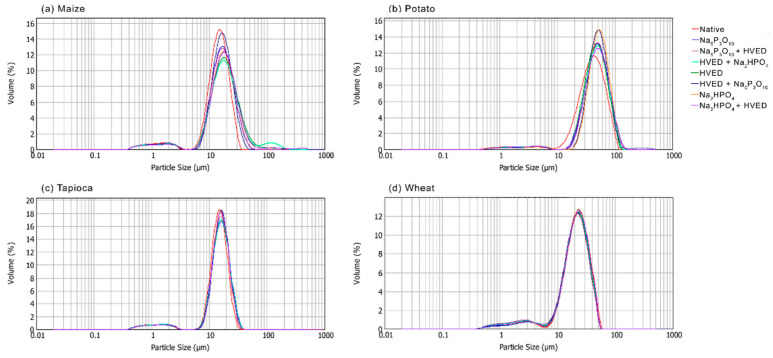
Particle size distribution of starches modified with high-voltage electrical discharge and phosphorylation.

The results of the particle size analysis are shown in the Figure 5. Svihus et al. [34] stated that the particle size determines enthalpy of gelatinization, and Singh et al. [35] linked this property to the paste clarity. The starches in this research indeed show a positive correlation of the particle size with gelatinization enthalpy (shown in the previous paper [9]). The decrease of the particle size after modifications of maize and wheat starches is visible from the presented results, while potato and tapioca starches were not significantly influenced regarding this property. The reduction of the particle size of the maize starch supports findings for the decrease of pasting temperature after the HVED treatment, proving the physical damaging of the granules by the HVED.

Namely, Okyere et al. [36], reviewing the influence of cold plasma treatment on starches of different origin, concluded that volatilization of starch surface (plasma etching) occurred due to surface bombarding by highly energetic species produced by plasma generation. Cracks, cavities, and fissures may also be formed, along with the decrease of crystallinity, and even starch depolymerization [36].

## 4. Conclusions

In the conditions applied in this research, high-voltage electrical treatment (HVED) induced lower contents of P in modified starches, but also had an effect on analyzed properties, both when it was conducted prior and after the chemical modification, reducing the influence of Na_5_P_3_O_10_ and Na_2_HPO_4_ on the decrease of pasting temperature. With minor exceptions, the gel strength of starches increased, and the rupture strength decreased by all modifications. HVED treatment resulted in a decrease of the particle size after the modification of maize and wheat starches, while potato and tapioca starches were not significantly influenced by the treatment.

The HVED treatment may be used as a tool in phosphorylation of starch, but care must be taken in adjusting the process to the starch type and a possible combination with chemical modifications. Although this research showed a limited influence of the HVED on the selected properties of starch (pasting, swelling power, solubility, texture), an application of different frequencies, times, and concentrations of starches during treatment could result in a more pronounced effect. In addition, when combining the HVED with chemical modifications, one has to bear in mind that the HVED may reduce the effect of a chemical modification on some starch properties.

## Figures and Tables

**Table 1:** Pasting properties of native and modified starches.

Starch	Treatment	Pasting Temperature (°C)	Maximum Viscosity (BU)	Gelatinisation Maximum (°C)	Viscosity at 92 °C (BU)	Viscosity after 15 min at 92 °C (BU)	Viscosity at 50 °C (BU)	Viscosity after 15 min at 50 °C (BU)	*Breakdown*	*Setback*
Maize	Native	73.35 ± 0.07 ^d^	258.50 ± 3.54 ^a^	88.40 ± 0.14 ^f^	247.00 ± 2.83 ^a^	167.00 ± 1.41 ^a^	327.50 ± 4.95 ^a.b^	300.50 ± 3.54 ^c^	91.50 ± 2.12 ^a^	160.50 ± 0.54 ^b^
	HVED	72.00 ± 0.14 ^c^	317.00 ± 9.90 ^b^	83.50 ± 0.42 ^e^	295.50 ± 7.78 ^b^	183.00 ± 5.66 ^a.b^	387.50 ± 0.71 ^b^	349.50 ± 9.19 ^e^	134.00 ± 4.24 ^b^	204.50 ± 4.95 ^c^
	Na_5_P_3_O_10_	68.25 ± 0.21 ^a^	562.00 ± 1.41 ^e^	71.05 ± 0.49 ^a^	429.00 ± 1.41 ^d.e^	263.50 ± 0.71 ^c^	525.50 ± 0.71 ^c^	448.50 ± 3.54 ^f^	298.50 ± 0.71 ^e^	262.00 ± 1.41 ^d^
	HVED + Na_5_P_3_O_10_	70.10 ± 0.00 ^b^	507.00 ± 1.41 ^d^	78.10 ± 0.00 ^c^	410.50 ± 2.12 ^d^	252.00 ± 1.41 ^b.c^	514.50 ± 0.71 ^c^	441.00 ± 2.83 ^f^	254.00 ± 2.83 ^d^	262.50 ± 2.12 ^d^
	Na_5_P_3_O_10_ + HVED	68.65 ± 0.07 ^a^	559.50 ± 0.71 ^e^	74.45 ± 0.07 ^b^	445.00 ± 11.31 ^e^	270.00 ± 1.41 ^a^	539.00 ± 4.24 ^c^	459.50 ± 2.12 ^f^	289.50 ± 0.71 ^e^	269.00 ± 2.83 ^d^
	Na_2_HPO_4_	72.30 ± 0.14 ^c^	389.00 ± 7.07 ^c^	80.30 ± 0.99 ^d^	329.00 ± 11.31 ^c^	172.00 ± 1.41 ^a^	504.00 ± 212.13 ^c^	326.50 ± 4.95 ^d^	217.00 ± 8.49 ^c^	182.00 ± 1.41 ^b^
	HVED + Na_2_HPO_4_	73.50 ± 0.14 ^d^	323.50 ± 3.54 ^b^	83.30 ± 0.57 ^e^	277.00 ± 4.24 ^b^	132.00 ± 7.07 ^a^	250.50 ± 4.95 ^a^	237.50 ± 4.95 ^a^	191.50 ± 10.61 ^c^	118.50 ± 2.12 ^a^
	Na_2_HPO_4_ + HVED	73.30 ± 0.42 ^d^	330.00 ± 7.07 ^b^	83.65 ± 0.35 ^e^	291.50 ± 0.71 ^b^	143.50 ± 7.78 ^a^	280.00 ± 9.90 ^a^	264.00 ± 8.49 ^b^	186.00 ± 14.14 ^c^	136.50 ± 2.12 ^a^
Wheat	Native	67.55 ± 0.49 ^B^	328.00 ± 2.83 ^B^	93.30 ± 0.85 ^A^	324.00 ± 1.41 ^C^	234.50 ± 2.12 ^B^	541.50 ± 6.36 ^C^	428.00 ± 7.07 ^C^	93.50 ± 0.71 ^A^	307.00 ± 4.24 ^c^
	HVED	68.25 ± 0.07 ^B^	321.00 ± 4.24 ^B^	92.05 ± 0.78 ^A^	320.00 ± 2.83 ^C^	236.50 ± 2.12 ^B^	549.00 ± 1.41 ^C^	447.00 ± 12.73 ^C^	84.50 ± 6.36 ^A^	312.50 ± 3.54 ^c.d^
	Na_5_P_3_O_10_	63.45 ± 0.49 ^A^	523.50 ± 2.12 ^C^	92.60 ± 0.42 ^A^	500.00 ± 21.21 ^D^	344.50 ± 10.61 ^C^	655.00 ± 18.38 ^D^	524.50 ± 13.44 ^D^	178.50 ± 7.78 ^B^	310.50 ± 7.78 ^c^
	HVED + Na_5_P_3_O_10_	62.50 ± 0.71 ^A^	521.00 ± 2.83 ^C^	92.75 ± 0.49 ^B^	510.00 ± 5.66 ^D.E^	348.00 ± 2.83 ^C^	680.50 ± 3.54 ^D^	553.50 ± 0.71 ^E^	173.00 ± 0.00 ^B^	332.50 ± 0.71 ^d^
	Na_5_P_3_O_10_ + HVED	62.80 ± 0.57^A^	540.50 ± 3.54 ^C^	93.10 ± 0.14 ^A^	527.50 ± 6.36 ^E^	352.50 ± 7.78 ^C^	669.00 ± 8.49 ^D^	540.00 ± 5.66 ^D.E^	186.50 ± 12.02 ^B^	316.50 ± 0.71 ^c.d^
	Na_2_HPO_4_	68.05 ± 0.35 ^B^	240.00 ± 12.73 ^A^	94.25 ± 1.48 ^A^	236.00 ± 7.07 ^B^	168.50 ± 4.95 ^A.B^	319.00 ± 7.07 ^B^	306.50 ± 9.19 ^B^	70.50 ± 7.78 ^A^	150.50 ± 2.12 ^b^
	HVED + Na_2_HPO_4_	70.80 ± 0.99 ^C^	209.50 ± 2.12 ^A^	93.35 ± 0.07 ^A^	194.00 ± 1.41 ^A^	125.00 ± 1.41 ^A^	229.50 ± 4.95 ^A^	220.50 ± 4.95 ^A^	85.00 ± 0.00 ^A^	104.50 ± 3.54 ^a^
	Na_2_HPO_4_ + HVED	70.65 ± 0.07 ^C^	219.00 ± 2.83 ^A^	93.15 ± 0.07 ^A^	205.50 ± 4.95 ^A^	142.00 ± 1.41 ^A^	251.00 ± 4.24 ^A.B^	240.00 ± 2.83 ^A^	76.50 ± 3.54 ^A^	109.00 ± 5.66 ^a^
Potato	Native	58.55 ± 0.21 ^b.c.d^	1640.00 ± 73.54 ^e^	65.05 ± 0.35 ^b^	698.50 ± 10.61 ^c^	514.50 ± 2.12 ^b^	1143.50 ± 10.61 ^c^	1011.00 ± 7.07 ^d^	1125.00 ± 76.37 ^d^	629.00 ± 8.49 ^e^
	HVED	60.40 ± 0.00 ^e^	1677.00 ± 29.70 ^f^	63.65 ± 1.34 ^b^	688.00 ± 7.07 ^c^	525.00 ± 1.41 ^b^	1149.00 ± 7.07 ^c^	1000.00 ± 15.56 ^d^	1151.50 ± 27.58 ^d^	624.00 ± 5.66 ^e^
	Na_5_P_3_O_10_	56.25 ± 0.21 ^a^	1509.00 ± 32.53 ^d^	67.35 ± 0.78 ^c^	862.50 ± 3.54 ^e^	496.00 ± 5.66 ^b^	885.50 ± 38.89 ^b^	731.50 ± 30.41 ^c^	1012.50 ± 26.16 ^c^	389.50 ± 33.23 ^d^
	HVED + Na_5_P_3_O_10_	58.50 ± 0.99 ^b.c^	1893.00 ± 11.31 ^h^	61.00 ± 0.00 ^a^	860.00 ± 43.84 ^e^	480.00 ± 19.80 ^b^	835.00 ± 15.56 ^b^	728.00 ± 16.97 ^c^	1413.00 ± 8.49 ^f^	355.00 ± 35.36 ^c^
	Na_5_P_3_O_10_ + HVED	55.90 ± 0.57 ^a^	1755.00 ± 26.87 ^g^	60.80 ± 0.28 ^a^	824.00 ± 0.00 ^d^	490.50 ± 0.71 ^b^	855.00 ± 22.63 ^b^	716.50 ± 17.68 ^c^	1262.00 ± 25.46 ^e^	364.50 ± 21.92 ^c^
	Na_2_HPO_4_	57.65 ± 0.35 ^b^	705.00 ± 16.97 ^c^	72.25 ± 2.62 ^d^	567.50 ± 12.02 ^b^	278.50 ± 4.95 ^a^	529.00 ± 7.07 ^a^	409.00 ± 4.24 ^b^	426.50 ± 12.02 ^b^	250.50 ± 2.12 ^b^
	HVED + Na_2_HPO_4_	58.90 ± 0.14 ^c.d^	579.00 ± 1.41 ^a^	74.90 ± 2.26 ^e^	513.50 ± 7.78 ^b^	243.00 ± 11.31 ^a^	454.00 ± 22.63 ^a^	371.50 ± 20.51 ^a^	335.00 ± 9.90 ^a^	211.00 ± 11.31 ^a^
	Na_2_HPO_4_ + HVED	59.50 ± 0.28 ^d.e^	617.00 ± 16.97 ^b^	81.05 ± 0.64 ^f^	555.00 ± 5.66 ^a^	274.00 ± 0.00 ^a^	517.00 ± 12.73 ^a^	419.50 ± 0.71 ^b^	342.00 ± 16.97 ^a^	243.00 ± 12.73 ^b^
Tapioca	Native	66.30 ± 0.57 ^B^	644.00 ± 0.00 ^C^	74.65 ± 0.92 ^B.C^	382.00 ± 2.83 ^B^	205.50 ± 0.71 ^A^	444.50 ± 6.36 ^C^	411.50 ± 3.54 ^C^	438.50 ± 0.71 ^C^	239.00 ± 5.66 ^c^
	HVED	66.10 ± 0.14 ^B^	648.00 ± 15.56 ^C^	74.45 ± 0.21 ^B.C^	387.50 ± 2.12 ^B^	205.00 ± 7.07 ^A^	422.50 ± 6.36 ^B.C^	409.50 ± 2.12 ^C^	442.50 ± 9.19 ^C^	217.50 ± 0.71 ^c^
	Na_5_P_3_O_10_	64.15 ± 0.07 ^A^	882.00 ± 1.41 ^D^	73.65 ± 0.07 ^A.B^	546.00 ± 7.07 ^D^	343.00 ± 0.00 ^B^	568.50 ± 9.19 ^D^	507.00 ± 2.83 ^E^	537.00 ± 1.41 ^E^	225.50 ± 9.19 ^c^
	HVED + Na_5_P_3_O_10_	65.05 ± 1.06 ^A^	805.50 ± 10.61 ^E^	73.60 ± 1.27 ^A.B^	482.00 ± 5.66 ^C^	317.50 ± 2.12 ^B^	584.50 ± 6.36 ^D^	481.50 ± 4.95 ^D^	488.00 ± 9.90 ^D^	267.00 ± 4.24 ^d^
	Na_5_P_3_O_10_ + HVED	64.40 ± 0.85 ^A^	920.50 ± 7.78 ^F^	72.45 ± 0.21 ^A^	556.00 ± 4.24 ^D^	492.50 ± 195.87 ^C^	619.00 ± 4.24 ^D^	527.50 ± 12.02 ^E^	562.00 ± 12.73 ^E^	261.50 ± 0.71 ^d^
	Na_2_HPO_4_	67.85 ± 0.64 ^C^	525.00 ± 2.83 ^B^	76.70 ± 0.28 ^D^	378.50 ± 4.95 ^B^	188.00 ± 1.41 ^A^	361.00 ± 1.41 ^A.B^	296.00 ± 4.24 ^B^	337.50 ± 0.71 ^B^	173.00 ± 0.00 ^b^
	HVED + Na_2_HPO_4_	69.35 ± 0.21 ^D^	428.00 ± 1.41 ^A^	76.95 ± 0.21 ^D^	314.50 ± 0.71 ^A^	151.50 ± 2.12 ^A^	295.50 ± 4.95 ^A^	238.50 ± 4.95 ^A^	275.50 ± 0.71 ^A^	144.00 ± 2.83 ^a^
	Na_2_HPO_4_ + HVED	69.25 ± 0.07 ^D^	445.00 ± 5.66 ^A^	76.15 ± 1.20 ^C.D^	325.50 ± 2.12 ^A^	157.00 ± 1.41 ^A^	308.50 ± 4.95 ^A^	250.00 ± 1.41 ^A^	288.50 ± 6.36 ^A^	151.50 ± 3.54 ^a.b^

BU, Brabender Units; Values with different superscripts in the same column are different for the same starch type (*p* < 0.05).

## Data Availability

The data presented in this study are available on request from the corresponding author.
